# CCT6A and CHCHD2 Are Coamplified with EGFR and Associated with the Unfavorable Clinical Outcomes of Lung Adenocarcinoma

**DOI:** 10.1155/2022/1560199

**Published:** 2022-07-28

**Authors:** Haiwei Wang, Xinrui Wang, Liangpu Xu, Yingying Lin, Ji Zhang

**Affiliations:** ^1^Fujian Maternity and Child Health Hospital, Fuzhou, Fujian, China; ^2^Ren-Ji Hospital, Shanghai Jiao Tong University School of Medicine, Shanghai, China; ^3^Rui-Jin Hospital, Shanghai Jiao Tong University School of Medicine, Shanghai, China

## Abstract

Chaperonin containing TCP1 subunit 6A (CCT6A) and coiled-coil-helix-coiled-coil-helix domain containing 2 (CHCHD2) are located at the chromosome 7p11 region proximal to epidermal growth factor receptor (EGFR). However, the amplifications, expressions, and the prognostic effects of CCT6A and CHCDH2 in lung adenocarcinoma (LUAD) are unclear. Here, using The Cancer Genome Atlas (TCGA) and Gene Expression Omnibus (GEO) datasets, we found that CCT6A was coamplified and coexpressed with EGFR in LUAD patients. CCT6A amplification was correlated with the unfavorable outcomes of LUAD. Moreover, CCT6A was upregulated in LUAD tissues, and CCT6A overexpression was correlated with the unfavorable relapse free survival or overall survival of LUAD. On the contrary, CCT6A was hypomethylated in LUAD, and CCT6A hypermethylation was correlated with the favorable overall survival of LUAD. Similar expression and methylation profiling of CCT6A were obtained in 479 lung normal tissues and 544 LUAD tissues collected from 11 independent datasets. In 1,462 LUAD patients from eight independent cohorts, CCT6A was also correlated with LUAD relapse-free survival or overall survival. Furthermore, CCT6A overexpression promoted the cell growth and invasion of LUAD. Identification of genes differentially expressed in CCT6A highly expressed LUAD patients revealed that CHCHD2 was the most correlated with CCT6A expression. CHCHD2 was coamplified with CCT6A. CHCHD2 was upregulated in LUAD tissues, and overexpression of CHCHD2 was correlated with the shorted relapse-free survival or overall survival of LUAD. Overall, our results revealed that CCT6A and CHCHD2 were coamplifying and coexpressing with EGFR and were correlated with the unfavorable clinical outcomes of LUAD.

## 1. Introduction

As a predominant subtype of non-small-cell lung cancer (NSCLC), the genetic variations of lung adenocarcinoma (LUAD) are extensively studied [1, 2]. Epidermal growth factor receptor (EGFR) is located at chromosome 7p11 region and is one driver alteration of LUAD [3]. Mutations or amplifications of EGFR leading to the constitutive kinase activations are required to sustain the uncontrolled proliferation of LUAD cells [4, 5]. Tyrosine kinase inhibitors (TKIs), like afatinib and gefitinib, are used in the standard treatment of LUAD harboring activating EGFR alterations and achieve superior clinical outcomes compared with chemotherapy [6–10]. Unfortunately, a number of LUAD patients inevitably develop resistance to EGFR-TKIs therapy [11]. EGFR T790M is the most frequent mutation, contributing to the acquired resistance to EGFR-TKIs [12–15]. Moreover, additional EGFR amplification is also correlated with the EGFR-TKI therapy response [16, 17] and the clinical overall survival of LUAD [18].

Except EGFR, chromosome 7p11 region includes multiple other genes. Neighboring genes proximal to EGFR regions may be coamplified with EGFR. Those coamplified genes are not just “passages” but also are suspected driver alterations to promote the progression of LUAD. For example, CHCHD2 is within 7p11 region and is coamplified with EGFR [19]. CHCHD2 is a mitochondrial protein [20] and is upregulated in lung cancer [21]. Moreover, CHCHD2 regulates the proliferation and migration of NSCLC cells and is associated with HIF1*α* expression [22]. Higher CHCHD2 or HIF1*α* expression is correlated with the worse clinical outcomes of NSCLC [23]. However, the expression and prognosis of CHCHD2 in LUAD are unclear.

CCT6A is another gene proximal to EGFR recurrent region and coamplified with EGFR [24]. CCT6A regulates the folding of cellular cytoskeletons and is correlated with the unfavorable clinical outcomes of cervical squamous cell carcinoma [25], colorectal adenocarcinoma [26], breast invasive carcinoma [27], and hepatocellular carcinoma [28]. In NSCLC, CCT6A is associated with TGF*β* downstream transcription factor SMAD2, and inhibition of CCT6A suppresses TGF*β*-mediated NSCLC metastasis [29]. CCT6A is also correlated with the lymph node metastasis and overall survival of NSCLC [30]. However, the expression, methylation, and the prognosis of CCT6A in NSCLC LUAD subtype remain largely unknown, and the associations between EGFR, CCT6A, and CHCHD2 at expression levels are still unclear in LUAD.

In this study, using The Cancer Genome Atlas (TCGA) [31] and Gene Expression Omnibus (GEO) datasets, we comprehensively analyzed the expression and methylation levels of CCT6A and CHCHD2. The prognostic effects of CCT6A and CHCHD2 in LUAD were also studied. Our results suggested that CCT6A and CHCHD2 were served as prognostic markers of LUAD.

## 2. Materials and Methods

### 2.1. Collection of TCGA and GEO Datasets

TCGA-LUAD datasets were collected from TCGA hub site [31]. The GSE7670 [32], GSE10072 [33], GSE27262 [34], GSE31908, GSE32665 [35], GSE43458 [36], GSE63459 [37], GSE75037 [38], GSE32867 [39], GSE63384 [37], and GSE62948 [40] datasets containing lung normal tissues and LUAD tissues were collected from GEO repositories. MSKCC datasets were previously published [41]. The GSE8894 [42], GSE30219 [43], GSE37745 [44], GSE42127 [45], GSE50081 [46], GSE68465 [47], and GSE72094 [48] datasets containing LUAD tissues and corresponding clinical survival information were also collected from GEO repositories. All datasets were analysis using R software.

### 2.2. EGFR, CCT6A and CHCHD2 Genetic Amplifications

Cooccurrence of CCT6A and EGFR amplification along with the cooccurrence of CCT6A and CHCHD2 amplification were downloaded from Cbioportal using TCGA-LUAD dataset [49, 50].

### 2.3. Univariate Cox Regression Analysis

The prognosis of CCT6A and CHCHD2 in LUAD was calculated using univariate cox regression analysis in R software “survival” package. The forest plots were generated using R software “forestplot” package.

### 2.4. Clinical Survival Analysis

The prognosis of CCT6A amplification was downloaded from Cbioportal. The prognostic values of CCT6A expression, CCT6A methylation, and CHCHD2 expression were tested using R software “survminer” package. LUAD patients were classified into “high” or “low” subgroups using the best cutoff points. *P* values were calculated by a log-rank test.

### 2.5. Overexpression of CCT6A in LUAD Cells

A549 and H1975 LUAD cells cultured in minimum Eagle's medium (MEM) with 10% fetal bovine serum (FBS) were used to determine the functions of CCT6A in LUAD by CCT6A overexpression. Briefly, cDNA of CCT6A was transfected into A549 or H1975 LUAD cells using lipofectamine 2000; then, the CCT6A overexpression was tested by western blot using CCT6A antibody.

### 2.6. Cell Proliferation CCK-8 Assay

CCK-8 assay was used to determine the cell proliferation after CCT6A overexpression. A549 and H1975 cells were cultured with 10 *μ*L CCK-8 in a 96-well plate. After 48 h, the cell proliferation was tested.

### 2.7. Wound Healing and Transwell Invasion Assay

A549 and H1975 cells were scratched using tips. After 24 h, the wound areas were determined with or without CCT6A overexpression. A549 and H1975 cells were cultured with 1% FBS in the upper chamber covered with Matrigel. The migrated A549 or H1975 was counted after 24 h.

### 2.8. Correlation Analysis

Correlation coefficients of CCT6A and CHCHD2 expression were calculated by spearman's correlation and demonstrated using R software “corrplot” package.

### 2.9. Statistical Analysis

Two-tailed paired Student's *t*-test was used to determine the different expression and methylation levels of CCT6A and CHCHD2 in LUAD. *P* value < 0.05 indicated the significantly difference.

## 3. Results

### 3.1. CCT6A Is Coamplified with EGFR, and CCT6A Amplification Is Correlated with the Poor Outcomes of LUAD

**Figure 1:**
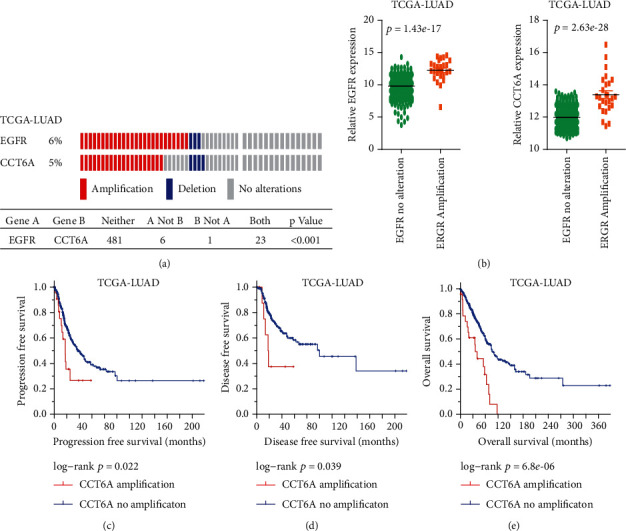
CCT6A is coamplified with EGFR, and CCT6A amplification is correlated with the poor outcomes of LUAD. (a) Cooccurrence of CCT6A and EGFR amplification in TCGA-LUAD. (b) Expression levels of EGFR and CCT6A in TCGA-LUAD patients without or with EGFR amplifications. Progression-free survival (c), disease-free survival (d), and overall survival (e) of LUAD patients without or with CCT6A amplification were determined using TCGA-LUAD dataset. *P* values were calculated by a log-rank test.

EGFR and CCT6A are both located at the chromosome 7p11 region [24]. We first tested the CCT6A and EGFR cooccurrence in LUAD. In 511 TCGA-LUAD patients, EGFR copy number variations were detected in 29 patients, while CCT6A copy number variations were detected in 24 LUAD patients. All LUAD patients with CCT6A amplifications were also with EGFR amplifications (Figure 1(a)). The coamplification of EGFR and CCT6A was statistically significant (Figure 1(a)). Corresponding with the EGFR amplification, the mRNA level of EGFR was increased in EGFR amplified LUAD (Figure 1(b)). Also, compared with EGFR-unamplified patients, CCT6A was highly expressed in EGFR-amplified LUAD (Figure 1(b)).

EGFR amplification was associated with the LUAD overall survival [51]. Similarly, LUAD with CCT6A amplification had poor progression-free survival contrast with LUAD without CCT6A amplification (Figure 1(c)). Moreover, LUAD tumor patients without CCT6A amplification had prolonged disease-free survival (Figure 1(d)) and overall survival (Figure 1(e)).

### 3.2. CCT6A Is Overexpressed in LUAD, and CCT6A Overexpression Is Correlated with the Poor Clinical Outcomes of LUAD

**Figure 2:**
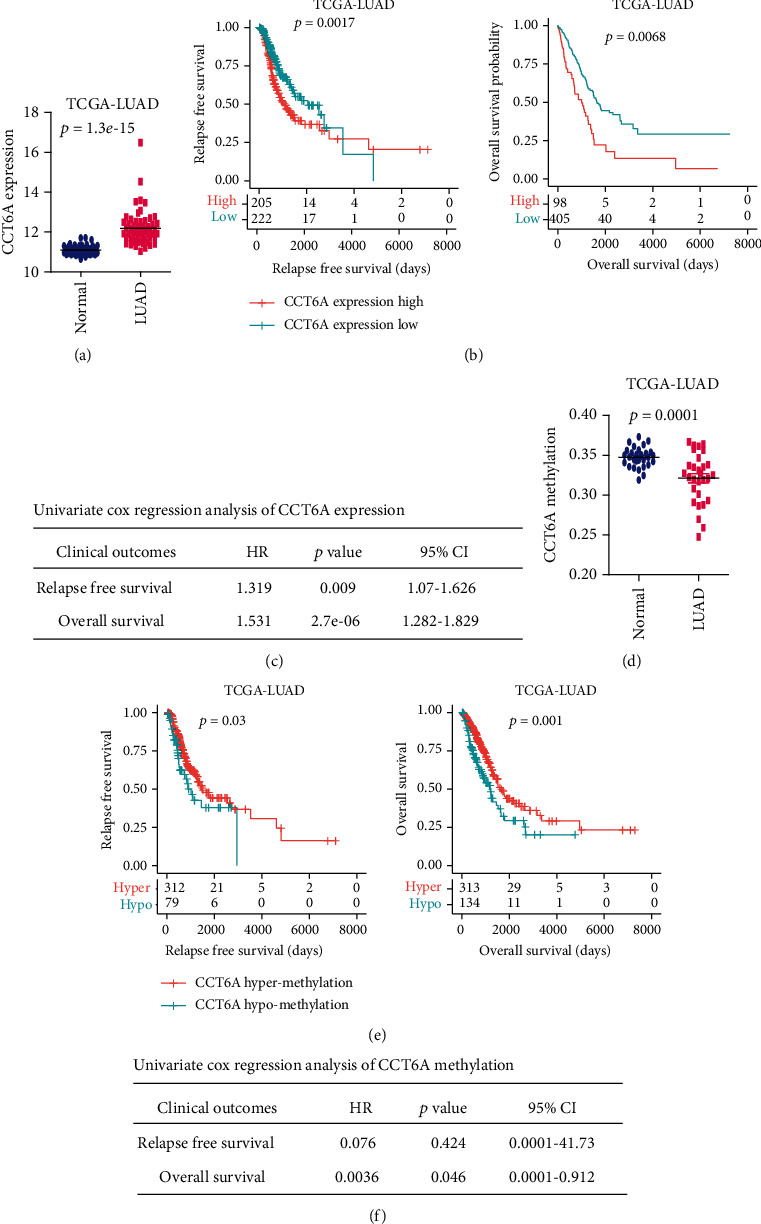
Expression, methylation, and prognosis of CCT6A in TCGA-LUAD dataset. (a) CCT6A expressions in LUAD and paired lung normal tissues based on TCGA dataset. (b) The relapse-free survival or overall survival of LUAD patients with CCT6A higher expressions or CCT6A lower expressions in TCGA dataset. *P* values were calculated using a log-rank test. (c) Associations of CCT6A expression levels with the relapse free survival or overall survival of LUAD in TCGA dataset. *P* values and hazard ratio (HR) were calculated by univariate cox regression. (d) CCT6A methylation levels in LUAD and paired lung normal tissues. (e) The relapse-free survival or overall survival of LUAD patients with hypermethylated CCT6A or hypomethylated CCT6A in TCGA dataset. (f) Associations of CCT6A methylation levels with the relapse-free survival or overall survival of LUAD in TCGA dataset.

Corresponding with the CCT6A amplification, mRNA levels of CCT6A were higher in LUAD, contrast with lung normal tissues (Figure 2(a)). Moreover, Kaplan-Meier survival analysis showed that LUAD patients with lower CCT6A expressions demonstrated prolonged relapse-free survival or overall survival compared with LUAD patients with higher CCT6A expressions in TCGA dataset (Figure 2(b)). Furthermore, univariate cox regression survival analysis confirmed that the expression of CCT6A was negatively correlated with the relapse-free survival or overall survival in TCGA-LUAD dataset (Figure 2(c)).

### 3.3. CCT6A Is Hypomethylated in LUAD, and CCT6A Hypomethylation Is Correlated with the Poor Clinical Outcomes of LUAD

Corresponding with the overexpression of CCT6A, the methylation of CCT6A was lower in LUAD contrast with lung normal tissues (Figure 2(d)). Moreover, LUAD with higher methylation levels of CCT6A had prolonged relapse-free survival and overall survival than LUAD with lower methylation levels of CCT6A in TCGA dataset (Figure 2(e)). Furthermore, in the univariate cox regression survival analysis, methylation of CCT6A was correlated with the overall survival of LUAD, but not associated with the relapse-free survival of LUAD in TCGA dataset (Figure 2(f)).

### 3.4. Validations of the Overexpression and Hypomethylation of CCT6A in LUAD Tissues Using GEO Datasets

Our results suggested that CCT6A was amplified and overexpressed in LUAD tissues and CCT6A methylation was lower in LUAD tissues. Moreover, CCT6A amplification and CCT6A overexpression were poor prognostic makers of LUAD, while CCT6A hypermethylation was associated with the favorable outcomes of LUAD in TCGA-LUAD dataset. Next, using LUAD cohorts from GEO datasets, we validated the expression, methylation, and prognosis of CCT6A in LUAD patients.

**Figure 3:**
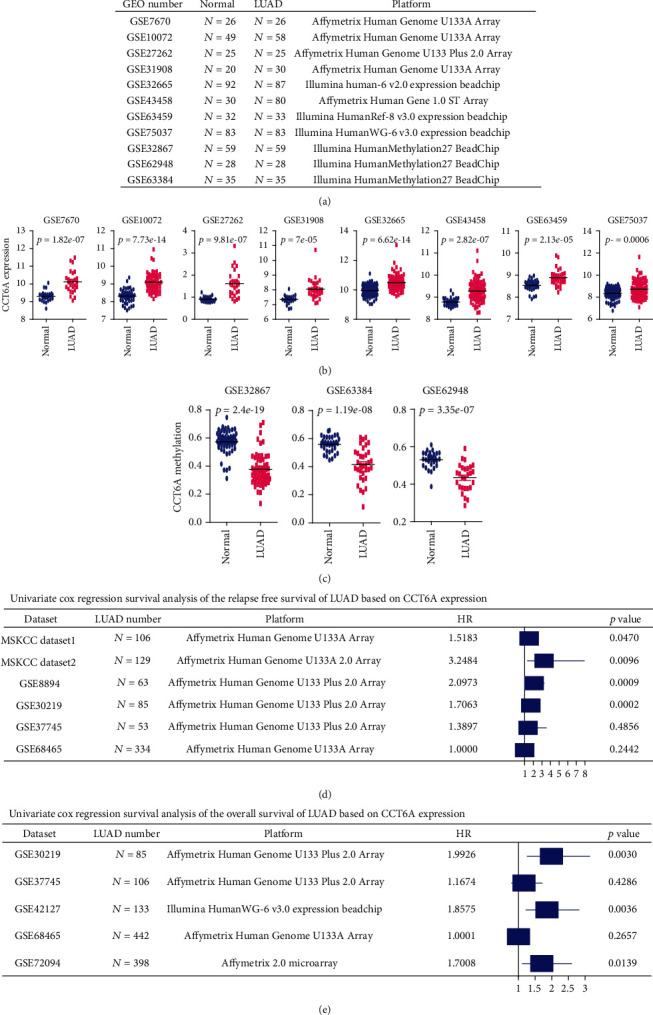
Expression, methylation, and prognosis of CCT6A in GEO-LUAD datasets. (a) A number of LUAD patients and platform of each GEO dataset used for the analysis of CCT6A expression and methylation. (b) The CCT6A expression levels in lung normal tissues and LUAD tissues in the GSE7670, GSE43458, GSE10072, GSE27262, GSE75037, GSE63459, GSE32665, and GSE31908 datasets. (c) The methylation levels of CCT6A in lung normal tissues and LUAD tissues in the GSE32867, GSE63384, and GSE62948 datasets. (d) Forest plot showed the associations of CCT6A with the relapse free survival of LUAD in MSKCC datasets and GSE8894, GSE30219, GSE37745, and GSE68465 datasets. A number of LUAD patients and platform of each GEO dataset were shown. Univariate cox regression analysis determined the HR and *P* values. (e) The associations of CCT6A with the overall survival of LUAD in the GSE68465, GSE30219, GSE42127, GSE37745, and GSE72094 datasets.

First, 357 lung normal tissues and 422 LUAD tissues were collected from 8 independent GEO datasets (Figure 3(a)). Similar results were obtained from the GSE7670, GSE10072, GSE27262, GSE31908, GSE32665, GSE43458, GSE63459, and GSE75037 datasets that the expression levels of CCT6A were higher in LUAD, compared with lung normal tissues (Figure 3(b)). Furthermore, DNA methylation of 122 lung normal tissues and corresponding LUAD tissues was collected from the GSE32867, GSE63384, and GSE62948 GEO datasets (Figure 3(a)). Consistent with TCGA-LUAD dataset, in the GSE32867, GSE63384, and GSE62948 datasets, methylation levels of CCT6A were lower in LUAD, contrast with lung normal tissues (Figure 3(c)).

### 3.5. Validations of the Prognosis of CCT6A in LUAD Patients Using MSKCC and GEO Datasets

In TCGA-LUAD dataset, expression and methylation levels of CCT6A were both correlated with the prognosis of LUAD. We then confirmed the prognostic significance of CCT6A using MSKCC and GEO datasets. Totally, 1,462 LUAD patients were collected from two MSKCC datasets, GSE8894, GSE30219, GSE37745, GSE68465, GSE42127, and GSE72094 six GEO datasets. Consistent with the results from TCGA-LUAD dataset, in two MSKCC datasets, GSE8894 and GSE30219 datasets, CCT6A was associated with the relapse-free survival of LUAD (Figure 3(d)). Moreover, CCT6A was associated with the overall survival of LUAD in the GSE42127, GSE72094, and GSE30219 GEO datasets (Figure 3(e)).

**Figure 4:**
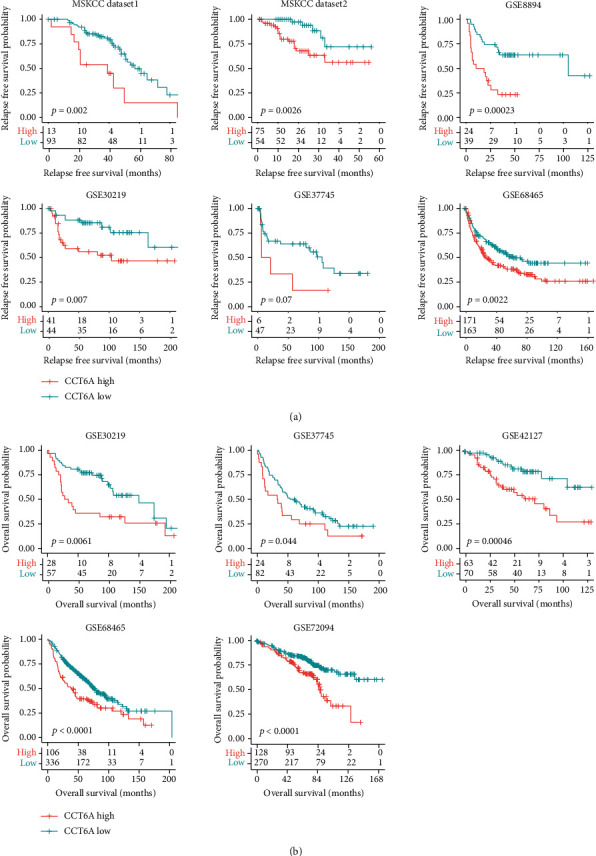
CCT6A higher expressions are associated with the LUAD lower relapse free survival or overall survival. (a) The associations between CCT6A expression and relapse-free survival in MSKCC datasets and GSE8894, GSE30219, GSE37745 and GSE68465 datasets. (b) The associations between CCT6A expression and LUAD overall survival in the GSE68465, GSE30219, GSE72094, GSE42127, and GSE37745 datasets.

The prognosis of CCT6A in LUAD was further analyzed using Kaplan-Meier survival analysis. CCT6A highly expressed LUAD had lower relapse-free survival than CCT6A lowly expressed LUAD in two MSKCC, GSE8894, GSE30219, GSE37745, and GSE68465 datasets (Figure 4(a)). CCT6A highly expressed LUAD also had shorted overall survival than CCT6A lowly expressed LUAD in the GSE30219, GSE37745, GSE42127, GSE68465, and GSE72094 GEO datasets (Figure 4(b)). All those results suggested the importance of CCT6A as an unfavorable marker in the prediction of the clinical outcomes of LUAD.

### 3.6. Overexpression of CCT6A Promotes the Growth and Invasion of LUAD

**Figure 5:**
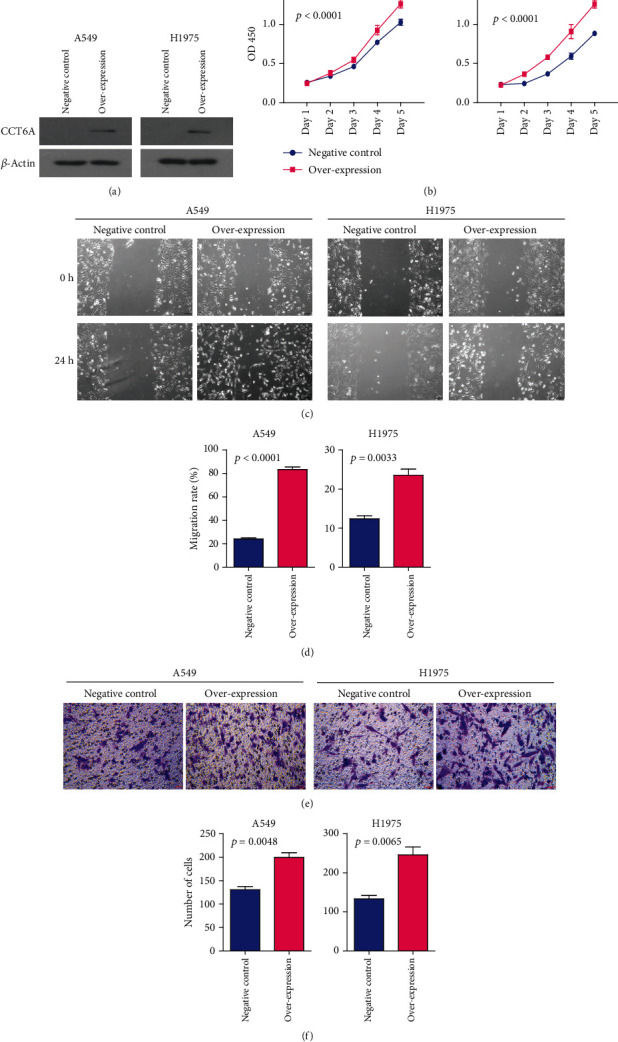
Overexpression of CCT6A promotes the cell growth and invasion of LUAD. (a) The overexpression of CCT6A in LUAD A549 and H1975 cell lines was validated by western blot. (b) CCK-8 assay was used to detect the growth of A549 and H1975 cells after CCT6A overexpression. (c) Representative images of the migration of LUAD A549 and H1975 cells after CCT6A overexpression in wound healing assay. (d) Statistical qualification of the migrated area in A549 and H1975 cells in wound healing assay. (e) Representative images of the invaded LUAD A549 and H1975 cells after CCT6A overexpression in transwell assay. (f) The number of invaded A549 and H1975 cells without or with CCT6A overexpression detected in transwell assay.

Next, biological functions of CCT6A in LUAD were determined. Two LUAD cell lines A549 and H1975 were overexpressed with CCT6A, as determined using western blot (Figure 5(a)). After CCT6A overexpression, the cell growth was significantly promoted in A549 and H1975 cells (Figure 5(b)). Moreover, overexpression of CCT6A increased the LUAD cell migration (Figure 5(c)). The migrated area was significantly increased after CCT6A overexpression in A549 and H1975 cells (Figure 5(d)). Furthermore, in transwell invasion assay, CCT6A overexpression significantly increased the invasive abilities of A549 and H1975 LUAD cells (Figure 5(e)). The invaded LUAD cells were increased by CCT6A overexpression (Figure 5(f)).

### 3.7. Identification of the Genes Associated with CCT6A

**Figure 6:**
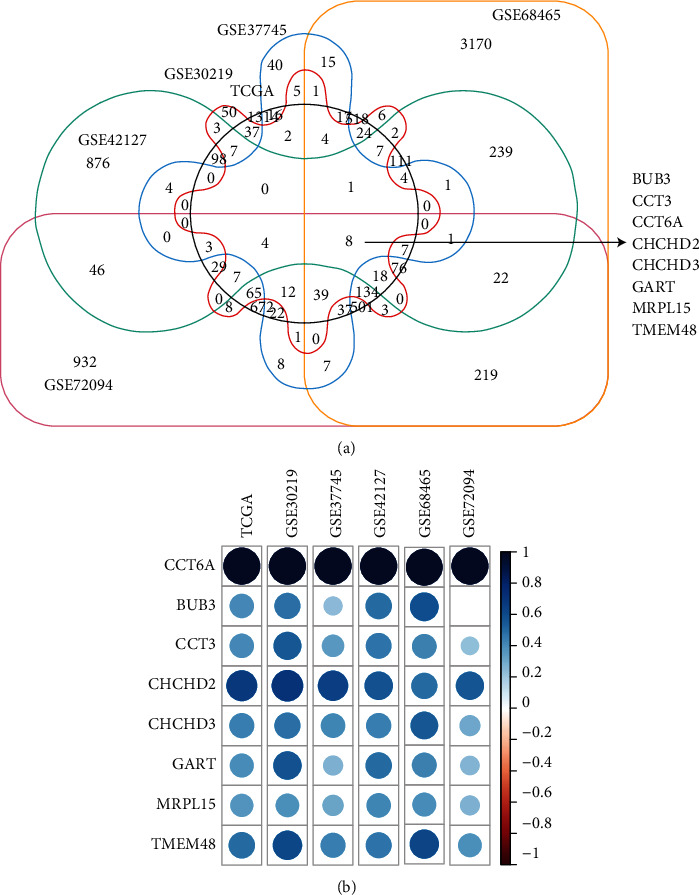
Identification of the genes associated with CCT6A. (a) Differentially expressed common genes between CCT6A highly expressed and CCT6A lowly expressed LUAD in TCGA, GSE37745, GSE30219, GSE42127, GSE72094, and GSE68465 datasets. (b) Corrplot demonstrated the correlations of CCT6A with BUB3, CCT3, CHCHD2, CHCHD3, GART, MRPL15, and TMEM48.

To further understand the prognosis and functions of CCT6A in LUAD, genes differentially expressed in LUAD with higher CCT6A expressions were identified in TCGA-LUAD, GSE72094, GSE42127, GSE30219, GSE37745, and GSE68465 datasets. Compared with LUAD patients with lower CCT6A expressions, 3794 genes were changed along with the higher CCT6A expressions in TCGA-LUAD dataset (Figure 6(a)). Moreover, 448, 259, 1574, 5192, and 2881 genes were significantly differently expressed in LUAD patients with higher CCT6A expressions in the GSE30219, GSE37745, GSE42127, GSE68465, and GSE72094 datasets, respectively (Figure 6(a)). Overlapping the differentially expressed genes showed that 8 genes, including CCT6A itself, were associated with the CCT6A expressions in TCGA-LUAD, GSE72094, GSE42127, GSE30219, GSE37745, and GSE68465 datasets (Figure 6(a)).

The correlation efficiency of CCT6A with BUB3, CCT3, CHCHD2, CHCHD3, GART, MRPL15, and TMEM48 expressions was further demonstrated. We found that CCT6A was the most correlated with CHCHD2 (Figure 6(b)).

### 3.8. CHCHD2 Is Overexpressed in LUAD, and CHCHD2 Overexpression Is Correlated with the Poor Clinical Outcomes of LUAD

**Figure 7:**
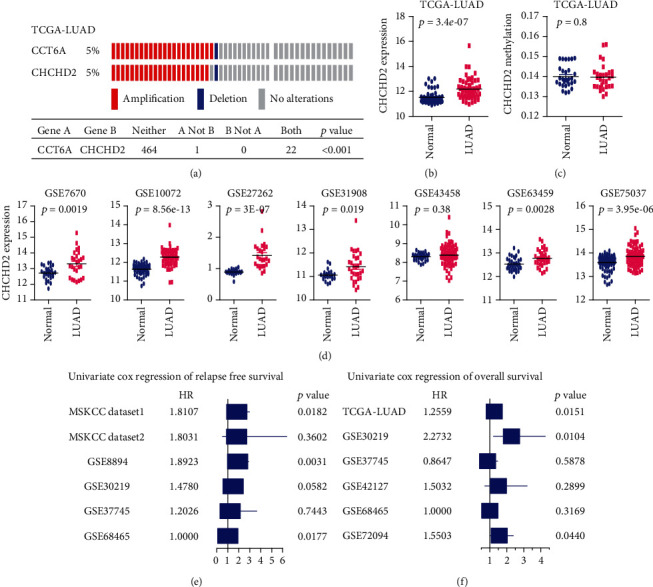
CHCHD2 is overexpressed in LUAD and CHCHD2 overexpression is correlated with the poor clinical outcomes of LUAD. (a) Cooccurrence of CCT6A and CHCHD2 amplification in TCGA-LUAD dataset. (b) Expression levels of CHCHD2 in lung normal and TCGA-LUAD tissues. (c) Methylation levels of CHCHD2 in lung normal and TCGA-LUAD tissues. (d) Expression levels of CHCHD2 in lung normal and LUAD tissues in GEO datasets. (e) Forest plot showed the associations of CHCHD2 with the relapse-free survival of LUAD in MSKCC datasets, GSE8894, GSE30219, GSE37745, and GSE68465 datasets. (f) Associations of CHCHD2 with the overall survival of LUAD in GSE37745, GSE30219, GSE68465, GSE42127, and GSE72094 datasets.

Like CCT6A, CHCHD2 is located at the chromosome 7p11 region, mapping proximal to EGFR gene. CHCHD2 was also coamplified with CCT6A in LUAD. All patients with CCT6A amplifications were also with CHCHD2 amplifications in TCGA-LUAD dataset (Figure 7(a)). CHCHD2 was higher in LUAD, contrast with lung normal tissues in TCGA-LUAD dataset (Figure 7(b)). However, the methylation levels of CHCHD2 in LUAD had no significantly difference from lung normal tissues (Figure 7(c)). Moreover, in the GSE7670, GSE63459, GSE10072, GSE31908, GSE27262, and GSE75037 datasets but not in the GSE43458 dataset, CHCHD2 was significantly highly expressed in LUAD (Figure 7(d)).

**Figure 8:**
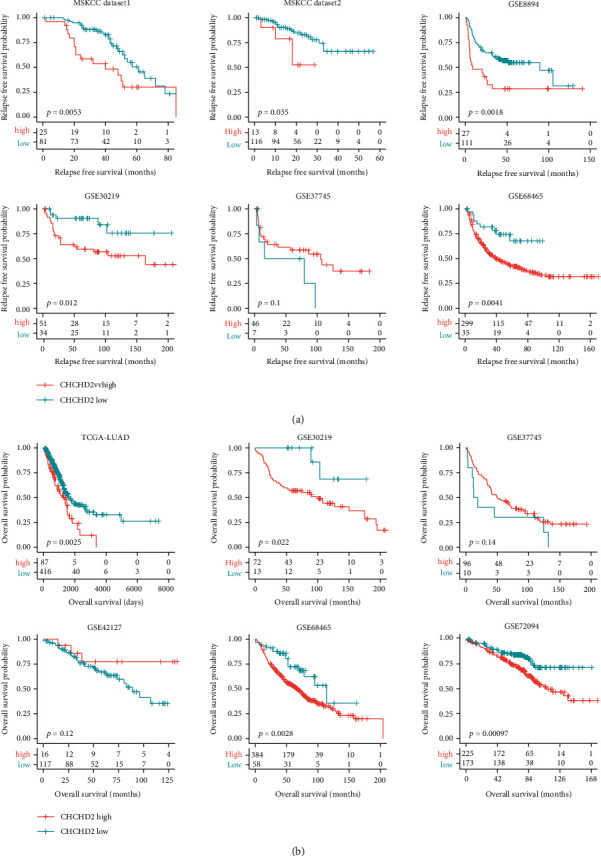
CHCHD2 higher expressions are correlated with the lower relapse-free survival and overall survival of LUAD. (a) Correlations between CHCHD2 and relapse-free survival of LUAD in MSKCC datasets and GSE8894, GSE30219, GSE37745, and GSE68465 datasets. (b) Correlations between CHCHD2 and overall survival of LUAD in the GSE37745, GSE30219, GSE68465, GSE42127, and GSE72094 datasets.

CHCHD2 was also a prognostic factor of LUAD. In MSKCC dataset1, GSE8894, and GSE68465 datasets, CHCHD2 was associated with the relapse-free survival of LUAD (Figure 7(e)). Moreover, CHCHD2 was correlated with the overall survival of LUAD in TCGA-LUAD, GSE30219, and GSE72094 datasets (Figure 7(f)). Furthermore, CHCHD2 highly expressed LUAD patients had lower relapse-free survival in two MSKCC datasets, GSE8894, GSE30219, and GSE68465 datasets, but not the in GSE37745 dataset (Figure 8(a)). CHCHD2 highly expressed LUAD patients had shored overall survival in TCGA-LUAD, GSE72094, GSE68465, and GSE30219 GEO datasets (Figure 8(b)). But in GSE37745 and GSE42127 datasets, CHCHD2 was not correlated with the overall survival of LUAD (Figure 8(b)).

### 3.9. Prognosis of CCT6A and CHCHD2 in Glioblastoma (GBM) and Lung Squamous Cell Carcinoma (LUSC)

**Figure 9:**
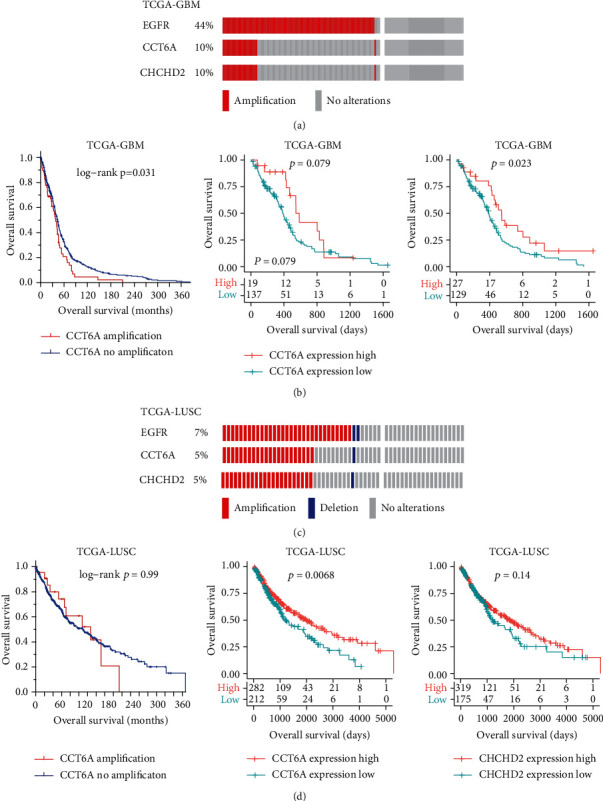
Prognosis of CCT6A and CHCHD2 in GBM and LUSC. (a) Cooccurrence of CCT6A, EGFR and CHCHD2 amplification in TCGA-GBM dataset. (b) Prognosis of CCT6A and CHCHD2 in TCGA-GBM patients. (c) Cooccurrence of CCT6A, EGFR, and CHCHD2 amplification in TCGA-LUSC dataset. (d) Prognosis of CCT6A and CHCHD2 in TCGA-LUSC patients.

Our previous results showed that EGFR was mostly amplified in patients with GBM and EGFR amplification represented an unfavorable prognostic factor of GBM [51]. In TCGA-GBM cohort, EGFR alteration was detected in 44% patients. CCT6A and CHCHD2 were significantly coamplified with EGFR in GBM (Figure 9(a)). Moreover, CCT6A amplification was correlated with the poor outcomes of GBM (Figure 9(b)). However, overexpression of CCT6A or CHCHD2 was not correlated with the poor outcomes of GBM (Figure 9(b)).

LUSC is another histological subtype of NSCLC with EGFR, CCT6A, and CHCHD2 coamplifications (Figure 9(c)). However, CCT6A amplification, CCT6A overexpression, or CHCHD2 overexpression was not associated with the shorted overall survival of LUSC (Figure 9(d)). Those results suggested the unique functions of CCT6A and CHCHD2 in the prognosis of LUAD.

## 4. Discussion

The gain of DNA copy number is a critical method of reactivation of oncogenes [52]. The amplified DNA regions can be as large as 1 Mb and encompass multiple genes. All genes in an amplicon are suspected tumor driver genes [53, 54]. Our analysis confirmed this hypothesis that CCT6A and CHCHD2 were coamplified with EGFR in the chromosome 7p11 region and CCT6A and CHCHD2 were both potential driver genes in the development of LUAD. Overexpression of CCT6A or CHCHD2 was associated with the progression of LUAD. Our results highlighted the critical roles of CCT6A and CHCHD2 in 7p11 amplicon in the development and progression of LUAD. Targeting of CCT6A or CHCHD2 may represent alternative therapeutic approaches for EGFR-amplified LUAD patients. Moreover, acquired resistance to EGFR-TKIs mediated by additional EGFR amplification could be alleviated by inhibitions of CCT6A or CHCHD2 expressions.

LUAD is extremely heterogeneous. Integrated analysis from different cohorts based on different gene expression technologies may provide the most robust results [55, 56]. In this study, by integrated analysis of TCGA, MSKCC, and GEO LUAD cohorts, we showed that CCT6A was overexpressed in LUAD and the overexpressions of CCT6A may be contributed by CCT6A amplification and CCT6A hypomethylation. Moreover, CCT6A amplification, CCT6A overexpression, and CCT6A hypomethylation were all correlated with the shorted relapse-free survival or overall survival of LUAD. Overexpression of CCT6A enhanced the growth and invasion of LUAD. So, from DNA copy number variations, mRNA expression, DNA methylation, and *in vitro* experiments, all our results suggested that CCT6A was an ideal prognostic maker of LUAD.

Among the differentially expressed genes in LUAD patients with higher CCT6A expressions, CHCHD2 was mostly correlated with CCT6A. CHCHD2 was also overexpressed in LUAD tissues and CHCHD2 overexpression was correlated with the shorted relapse-free survival or overall survival of LUAD. However, the methylation levels of CHCHD2 in LUAD were not different from lung normal tissues, suggesting that the gain of CHCHD2 DNA copy number was the main reason of upregulation of CHCHD2 in LUAD.

To our best knowledge, this is the first integrated analysis of the expression and prognosis of CCT6A and CHCHD2 in LUAD. Our results suggested that CCT6A and CHCHD2 served as prognostic biomarkers and potential therapeutic targets of LUAD. Although reports showed that higher CCT6A and CHCHD2 expressions were correlated with the worse clinical outcomes of NSCLC [30], the prognosis of CCT6A and CHCHD2 in NSCLC LUSC subtype was not significant, suggesting the unique functions of CCT6A and CHCHD2 in contribution of the development and progression of LUAD.

## 5. Conclusions

CCT6A and CHCHD2 were coamplifying and coexpressing with EGFR. CCT6A amplification was correlated with the shorted overall survival of LUAD. CCT6A and CHCHD2 were upregulated in LUAD. CCT6A and CHCHD2 overexpression was associated with the worse outcomes of LUAD. CC6TA was hypomethylated in LUAD tissues, and CCT6A hypermethylation was associated with the prolonged relapse-free survival or overall survival of LUAD.

## Data Availability

Previously reported datasets used in this study are available at TCGA, MSKCC, and GEO datasets. These datasets were cited at relevant places within the text.
